# Supplementary material for: Use of patient-centred outcome measures alongside the personal wheelchair budget process in NHS England: A mixed methods approach to exploring the staff and service user experience of using the WATCh and WATCh-Ad
Source: PLoS One. 2025 Jan 10;20(1):e0312967. doi: 10.1371/journal.pone.0312967 (PMC11723643; doi:10.1371/journal.pone.0312967)
Supplement: S2 File — (PDF) [file pone.0312967.s002.pdf]

WATCh

(Wheelchair outcomes Assessment Tool for Children)

Information for assessors and clinical staff

The aim of this form is to find out the aspects of life most important to the child or young adult who is being assessed for a wheelchair.

It should be completed at the assessment visit and the results kept with the patient records. It is intended that the user will be contacted again three to six months after receiving their chair, and asked to complete Part C (a follow-up survey) to see if the patient has experienced any positive changes to their life.

This process should help us improve our services by making sure we focus on users' key needs.

The form should be completed as far as possible by the child or young adult themselves, but in some circumstances they may need assistance from their parent/carer or yourself. Parents/carers are also allowed to complete this form on behalf of their child when the child is unable to do it themselves. We would suggest that you talk through the items in Part A with the user, and encourage them to describe what they want to achieve in Part B.

When sending out the Part C follow-up survey to a patient please ensure that the patient's previous 'top 5' from Part A/Part B have been transferred on to Part C.

An example of how the form should be filled out is presented on the next page.

Example of how to complete Part A

Area of your life	How your wheelchair could help	Top 5
1. Activities and fun	Help you to take part in activities and fun	✓
2. Independence	Help you to do more without help from other people	
3. Social life	Help you to spend time with your friends and family	
4. Moving around	Help you to get around inside and outside of the house	✓
5. Pain and discomfort	Help to reduce your pain or discomfort related to posture	
6. Self-care	Help you to wash and dress yourself	
7. Feeling included	Help you to feel part of wider society	
8. Managing your condition	Help to manage your condition and avoid health problems	✓
9. Communication	Help you to communicate and interact with others	
10. Education	Help you to go to school and learn	
11. Happiness	Help you to feel happy and free from worry	✓
12. Safety	Help you to feel safe and secure	
13. Parent or carer wellbeing	Help your parent or carer to stay happy and healthy	
14. Self-esteem and confidence	Help you to feel more self-confident	
15. Energy and fatigue	Help you to feel more energetic and less tired	✓
16. Achievement and goals	Help you to achieve the things that are important to you	

Patient ticks their top FIVE areas

Example of how to complete Part B/Part C

Top 5 (in order)	Area	What you want to achieve or feel
1 (most important)	*Energy and fatigue (no.15)*	*Feel less tired when using my wheelchair*
2	*Moving around (no.4)*	*Be able to move around school by myself*
3	*Happiness (no.11)*	*Feel less worried and upset*
4	*Activities and fun (no.1)*	*Be able to go to the shops with friends*
5 (less important)	*Pain and discomfort (no.8)*	*Improve my posture and reduce pain*

Answers transferred from Part A

How satisfied or happy you are with this area of your life

Top 5	Very Dissatisfied	Dissatisfied	Neutral	Satisfied	Very Satisfied
1		X			
2				X	
3			X		
4	X				
5		X			

WATCh Assessment Form

(Wheelchair outcomes Assessment Tool for Children)

Information for wheelchair users and parents/carers

We are using this form as part of your assessment, to help us to find out what goals you have in relation to your wheelchair. The form has two parts:

- Part A lists some areas of your life which your wheelchair might be able to help you with. Please decide which are the **FIVE** most important areas to you
- Part B then asks you to score how satisfied or happy you are now with each of the top **FIVE** areas you chose in Part A

Once you've had your wheelchair for a few months, we will ask you to score your top five list again to see if there has been any improvements. If you have any questions about the form, or problems filling it in, please let the person doing your assessment know. See below for an example of how to complete this form.

Example of how to complete Part A

Area of your life	How your wheelchair could help	Top 5
1. Activities and fun	Help you to take part in activities and fun	✓
2. Independence	Help you to do more without help from other people	
3. Social life	Help you to spend time with your friends and family	
4. Moving around	Help you to get around inside and outside of the house	✓
5. Pain and discomfort	Help to reduce your pain or discomfort related to posture	
6. Self-care	Help you to wash and dress yourself	
7. Feeling included	Help you to feel part of wider society	
8. Managing your condition	Help to manage your condition and avoid health problems	✓

Tick your top FIVE areas

Example of how to complete Part B

Top 5 (in order)	Area	What you want to achieve or feel	Very Dissatisfied	Dissatisfied	Neutral	Satisfied	Very Satisfied
1 (most important)	*Energy and fatigue (no.15)*	*Feel less tired when using my wheelchair*		X			
2	*Moving around (no.4)*	*Be able to move around school by myself*				X	
3	*Happiness (no.11)*	*Feel less worried and upset*			X		
4	*Activities and fun (no.1)*	*Be able to go to the shops with friends*	X				
5	*Pain and discomfort (no.8)*	*Improve my posture and reduce pain*		X			

How satisfied or happy you are with this area of your life

Transfer answers from Part A

Patient name:____________________________DOB:_____/____/______ NHS No.:_________________

Service Reference: _______________Assessor: ___________________________________Date:____/____/____________

Completed by: *(please tick)* Patient ☐ Parent/Carer ☐ Assessor ☐

PART A

Below are a list of different areas of your life which your wheelchair could make a difference to. Please look at this list and place a tick in the box next to the top 5 most important areas for you. Please make sure you only chose FIVE areas. If there's something missing from the list, you can write it in the space at the bottom.

Area of your life	How your wheelchair could help	Top 5
1. Activities and fun	Help you to take part in activities and fun	
2. Independence	Help you to do more without help from other people	
3. Social life	Help you to spend time with your friends and family	
4. Moving around	Help you to get around inside and outside of the house	
5. Pain and discomfort	Help to reduce your pain or discomfort related to posture	
6. Self-care	Help you to wash and dress yourself	
7. Feeling included	Help you to feel part of wider society	
8. Managing your condition	Help to manage your condition and avoid health problems	
9. Communication	Help you to communicate and interact with others	
10. Education	Help you to go to school and learn	
11. Happiness	Help you to feel happy and free from worry	
12. Safety	Help you to feel safe and secure	
13. Parent or carer wellbeing	Help your parent or carer to stay happy and healthy	
14. Self-esteem and confidence	Help you to feel more self-confident	
15. Energy and fatigue	Help you to feel more energetic and less tired	
16. Achievement and goals	Help you to achieve the things that are important to you	
17. Anything else? Please tell us here:		

Patient name:________________________________DOB:____/____/______ NHS No.: ______________

Service Reference:___________________________ Assessor: _________________________________Date:____/____/__________

PART B

In the boxes below, please write your top 5 areas from Part A in the order of their importance. You can also add a bit more information about what you want to achieve. So, if you chose 'activities and fun' in your top 5, you could say what you want to achieve, like starting a new sport or hobby. Please then rate how satisfied or happy you are now with your experience of the 5 areas, on a scale from 'very dissatisfied' to 'very satisfied'. For instance, if you aren't happy with how much pain you have at the moment, you might tick 'dissatisfied'.

Top 5 (in order)	Area	What you want to achieve or feel
1 (most important)		
2		
3		
4		
5		

How satisfied or happy you are with this area of your life

Very Dissatisfied	Dissatisfied	Neutral	Satisfied	Very Satisfied
○	○	○	○	○
○	○	○	○	○
○	○	○	○	○
○	○	○	○	○
○	○	○	○	○

WATCh Follow-up Form

(Wheelchair outcomes Assessment Tool for Children)

Information for wheelchair users and parents/carers

You may remember that we gave you a form to complete before you got your wheelchair, to help us find out what goals you had in relation to your wheelchair.

In Part B of the form we asked you to tell us a bit more about the 'Top 5' most important areas of your life and to score how satisfied or happy you were with them before getting your wheelchair.

Now you have had your wheelchair for a little while, we would like you to score these 'Top 5' again to see if your wheelchair has helped.

If you have any questions about the questionnaire, or need help filling it in, please speak to your therapist or clinical team.

Patient name:__________________________________DOB:____/____/______ NHS No.:________________

Service Reference:______________________ Assessor: ___________________________________Date:____/____/_________

PART C Follow up

Below are the top 5 areas you chose when you filled in the questionnaire at your assessment. Now that you have had your wheelchair for a little while, please rate how satisfied or happy you are now with these 5 areas, on a scale from 'very dissatisfied' to 'very satisfied'. For instance, if you aren't happy with how much pain you have at the moment, you might tick 'dissatisfied'.

Top 5 (in order)	Area	What you wanted to achieve or feel	How satisfied or happy you are with this area of your life
1 (most important)			○ Very Dissatisfied ○ Dissatisfied ○ Neutral ○ Satisfied ○ Very Satisfied
2			○ Very Dissatisfied ○ Dissatisfied ○ Neutral ○ Satisfied ○ Very Satisfied
3			○ Very Dissatisfied ○ Dissatisfied ○ Neutral ○ Satisfied ○ Very Satisfied
4			○ Very Dissatisfied ○ Dissatisfied ○ Neutral ○ Satisfied ○ Very Satisfied
5			○ Very Dissatisfied ○ Dissatisfied ○ Neutral ○ Satisfied ○ Very Satisfied
