# Supplementary material for: Use of patient-centred outcome measures alongside the personal wheelchair budget process in NHS England: A mixed methods approach to exploring the staff and service user experience of using the WATCh and WATCh-Ad
Source: PLoS One. 2025 Jan 10;20(1):e0312967. doi: 10.1371/journal.pone.0312967 (PMC11723643; doi:10.1371/journal.pone.0312967)
Supplement: S3 File — (PDF) [file pone.0312967.s003.pdf]

WATCH / WATCh-Ad PWB Project:

Staff Survey

This page may be removed and kept for information.

About the WATCh/WATCH-Ad Tools

The WATCh (Wheelchair outcomes Assessment Tool for Children) and the related WATCH-Ad for adults tools are patient-centred outcome measures. These are designed to help providers understand what patients want to achieve from their healthcare, and to understand whether or not they have achieved their desired outcomes.

The WATCh/WATCh-Ad Tools are intended for completion by the patient/user (or their parent/carer) before or at the assessment visit for a wheelchair.

Instructions for use are at the front of each Tool but in summary: patients are asked to select their 'Top 5' outcomes from the options listed, provide some specific examples and rate their current level of satisfaction. They will then be asked to score themselves against these 'Top5' areas a few months afterwards, to see how well their desired outcomes have been achieved.

About the WATCh/WATCH-Ad PWB Project

NHS England would like to assess how use of the Tools could fit within the pathway for Personalised Wheelchair Budgets (PWBs) and have funded this study with Bangor University. It is seeking feedback from staff and patients. It has been approved by local ethics committees.

What will I have to do?

Your wheelchair service is one of the sites taking part and you will have received information from your managers on the process. The Assessment Tools should be used in any assessment visit with users (unless neither they nor any accompanying adult are able to understand English sufficiently).

This survey is seeking your views on the use of the Tool in practice. We are particularly interested in: how long it took for the users to complete it; who completed it (for example the user by themselves or the parent/carer or yourself); what problems arose (if any) and whether you were able to deal with any problems or questions.

We are also asking about how use of the Tool impacted on the PWB process. We would also like to have your views on any issues in practice, and further explanation or training required etc.

We would also like to contact a small number of staff who have experience of using the WATCh Tools to take part in a short (15 minute) telephone interview. With your permission this will be recorded and transcribed by the researchers and the information used in the analysis.

We will be providing consent forms relating to your participation in this study. Your views will be used to help develop future guidance, will be handled securely and treated in confidence. While we will not name you in any reporting without your permission, we may want to use quotations.

Thank you for taking the time to read this and to give your thoughts on using the WATCh/ WATCh-Ad tool.

If you have any questions, please speak to your manager or contact the researcher using the details overleaf

Contact Details for further information:

Dr Lorna Tuersley
Ardudwy Building,
Bangor University,
Bangor, Gwynedd, LL57 2PZ

Telephone: 01248 38 8721
Mobile: 07979 081697
Email: l.tuersley@bangor.ac.uk
Website: cheme.bangor.ac.uk

If you have any concerns or complaints about this project and would like to speak to someone outside of the research team, please contact: Kate Buffery, Personalised Care Senior Programme Manager, NHS England and NHS Improvement, Skipton House, 3D 80 London Road, London SE1 6LH Email: k.buffery@nhs.net

This project is funded by NHS England. It has been approved by an NHS ethics committee. It is sponsored by Bangor University in partnership with NHS England and NHS Improvement

Your initials: ____________________ Job title: ______________________________Grade: ________

1. Date of Consultation _____/________/_________ 2. Age of user __________ Years

3. How long did it take for the user to complete the WATCh or WATCh-Ad form? (Approximately)
___________________ minutes

4. Who completed the form?

Patient ☐ Parent/Carer ☐ You ☐ Patient with help ☐

5. Were there any problems or questions? If so please specify: **Yes/No**

Comment:

6. Were you able to deal with any problems or questions about the form? **Yes/No**
If not please specify

Comment:

7. Based on this experience, do you feel that use of the WATCh or WATCH-Ad tool was useful in the development of the personalised care plan? **Yes/No**

Comment:

8. Based on the health and wellbeing outcomes identified in the personalised care and support plan did this impact on prescription choice/type of wheelchair? **Yes/No**

Comment:

9. Which PWB deployment option was required in order to meet the identified health and wellbeing outcomes?

Notional ☐ | Notional with Contribution ☐ | Third Party ☐ | Not deployed ☐

10. How much extra (or less) time do you think was spent completing and managing the WATCh / WATCh-Ad Tool in addition to the Personal Wheelchair Budget discussions by:

You:_________________minutes **more** or _________________ minutes **less**

Other staff involved in the assessment (pleased state the type e.g. Role 1 engineer, Role 2 administrator etc.)

Role 1____________________________________Time: _________________minutes **more/less**

Role 2____________________________________ Time: _________________minutes **more/less**

Role 3____________________________________ Time: _________________minutes **more/less**

Role 4____________________________________ Time: _________________minutes more/less

Comment:

11. Thank you! Please add any other thoughts below, then put this form in the envelope provided and hand back to your local co-coordinator

Wheelchair User Survey
Please help us with the WATCh/WATCh-Ad PWB project!

Thank you for reading the information about the project.

This short survey is to help us find out what you thought about your visit today, in particular about the WATCh or WATCh-Ad Form asking you about the outcomes most important to you. We want to know how you found this and how you thought it could help alongside you being offered a Personal Wheelchair Budget or PWB. Anything you tell us will be treated in strictest confidence.

We need the views of as many wheelchair users as possible. You can fill in the form yourself, or a parent or carer can help if needed. When you have finished you can put your completed form in the envelope provided and give it to a member of clinic staff.

Please try to complete as much of the survey as you can. If you cannot, or do not want to answer a question, just leave it blank. If you need help to complete it, or don't understand anything, please ask your parent or carer. It is fine if you want someone else to complete the survey for you, but it is important that we have ~~**your**~~ views.

<u>Information for parents, carers or consultees</u>:
The questions have been written for the person who needs a wheelchair to complete by themselves wherever possible. You may complete for them or your child, or the person you are acting as consultee for on their behalf if they need or want this.

If they can answer the questions themselves but need help to fill in the form, including reading questions and writing answers, that is fine. If they do not understand a question, please repeat it to them and encourage them to answer it according to what they think it means. Try not to influence their answers.

Telephone Interviews

We would like to speak to some of the people who have filled in this survey in a short (15-20 minute) telephone call. This is to find out some more details about how they found the assessment. If you would like to be contacted about this, there is space on the survey to give us your contact details. If you do not wish to do this, that's fine, but do please complete the survey

If you have any questions please contact the research team:

Lorna Tuersley

Centre for Health Economics and Medicines Evaluation
Ardudwy, Bangor University
Gwynedd, LL57 2PZ

Telephone: 01248 38 8721 **Mobile:** 07792670053
Email: ~~l.tuersley@bangor.ac.uk~~ **Website:** cheme.bangor.ac.uk

WATCh/WATCH-Ad PWB SURVEY

Service ID ____________________

Part 1

Please read the following information. Put a tick in each box to tell us where you are saying ‘yes’ or leave blank the answer is ‘no’

I have read the information leaflet about the WATCh/WATCH-Ad PWB project. ☐

I understand what I am being asked to do. ☐

I know that my information and answers in this survey will be kept private. ☐

I know you will not use any names when you tell people what you found out in the project. ☐

I agree to allow the answers given to be used for educational purposes in the future. ☐

I agree to complete this survey as part of the WATCh/WATCh-Ad PWB project. ☐

I am happy for the research team to contact me about a telephone interview ☐

I understand that my future WATCh scores may be requested by Individuals from Bangor University where it is relevant to my taking part in this research. I give permission for this. ☐

Service ID ____________________

Part 2 – Completing the WATCh or WATCh-Ad Form

Q1 Who completed the WATCh or WATCH-Ad form?

You (the user) ☐ Parent/Carer ☐ Member of staff ☐ You with help ☐

Q2 How long did it take to complete the WATCh or WATCH-Ad Tool? __________ Minutes

Q3 Was it easy to understand what to do? Yes ☐ **No** ☐

Q4 Did you have any questions or problems? Yes ☐ **No** ☐

If yes, can you tell us about them here?

Q5 Did you think anything important was missing? Yes ☐ **No** ☐

Can you say something about that here?

Q6 How helpful was filling in the WATCh or WATCh-Ad tool in helping discuss your needs in the appointment?

Very helpful ☐ Quite helpful ☐ Not very helpful ☐ Not at all helpful ☐

Can you say something about that here?

Service Ref: ____________________

Q6 **How did this compare with any previous assessment you may have had where the Tool was not used?**

Better ☐ No different ☐ Worse ☐ Not had a previous assessment ☐

Can you say something about that here?

Q7 **After you have received any equipment based on this assessment, the wheelchair service will ask you to complete a WATCh or WATCh-Ad follow-up form asking about how well it has met the needs you discussed today.**

How would you feel about this?

r Happy to do this ☐ neither happy nor unhappy ☐ Not very happy to do this ☐

Can you say something about that here? For example the best way to contact you such as by letter or email or a phone call?

Q8 **Is there anything else you would like to tell us? For example, if you want to say more about any of your answers above, or you want to tell us anything else which might help to improve how the WATCh or WATCh-Ad tool is used in your visit.**

Can you say something about that here?

Service ID ____________________

Part 3

Information about you (or the user if you are completing for them)

Please place a tick in the box. If an answer needs writing in, please use BLOCK CAPITALS.

If you don't know the answer to a question, please leave it blank.

Q9 **Are you** Male ☐ Female ☐ Other (please specify) ☐ ________________ Prefer not to say ☐

Q10 **What is your age in years?** ☐ Prefer not to say ☐

Q11 **How long have you been using a wheelchair in years?**

Never used ☐ Less than 1 year ☐ 1-5 years ☐ 5-10 years ☐ More than 10 years ☐

Q13 **How long ago was your last assessment by the NHS for a wheelchair before this visit?**

This is the first time ☐ Less than 1 year ☐ 1-5 years ☐ 5-10 years ☐ More than 10 years ☐

Q 14 **Please tell us about any wheelchairs or other mobility equipment you use now. If you know the name, please add this. Tick the box if it is used indoors and/or outdoors and if it is powered.**

**Item of equipment (e.g. wheelchair, scooter)**	**Indoor**	**Outdoor**	**Powered**
**1)**________________________________	☐	☐	☐
**2)**________________________________	☐	☐	☐
**3)**________________________________	☐	☐	☐
**4)**________________________________	☐	☐	☐
**5)**________________________________	☐	☐	☐

Q15 **Can you tell us about your main reason for using a wheelchair?**

____

____

____

Part 4 – Telephone interview

If you are happy to be contacted about a telephone interview please make sure you have ticked the box in part 1 and let us have your details in the box below. These will be kept in strict confidence and destroyed at the end of the study.

You do not have to take part in an interview and your decision about whether to do this will not affect your care in any way.

Please also let us know how you would like to be contacted:

I am happy to be contacted about the interview (fill in details in the box below):

By phone ☐ In writing ☐ By email ☐

Contact details to arrange a telephone Interview

____

Name

Home address ____

____

Post code ____________________ Phone number __________________

Email address ____

If you are completing this on behalf of a child or on behalf of an adult unable to give consent:

__________________________ __________________________

Your name Your relationship to wheelchair user

Thank you for completing this survey!

Please put this into the envelope provided and hand back to a member of staff.

For further information on the study please contact:

Dr Lorna Tuersley, Centre for Health Economics and Medicines Evaluation
Ardudwy, Bangor University, Gwynedd, LL57 2PZ **Telephone:** 01248 38 8721
Email: l.tuersley@bangor.ac.uk **Website:** cheme.bangor.ac.uk
