# Supplementary material for: Use of patient-centred outcome measures alongside the personal wheelchair budget process in NHS England: A mixed methods approach to exploring the staff and service user experience of using the WATCh and WATCh-Ad
Source: PLoS One. 2025 Jan 10;20(1):e0312967. doi: 10.1371/journal.pone.0312967 (PMC11723643; doi:10.1371/journal.pone.0312967)
Supplement: S4 File — (PDF) [file pone.0312967.s004.pdf]

FLOWCHART WATCh PWB Supplementary file 4.docx

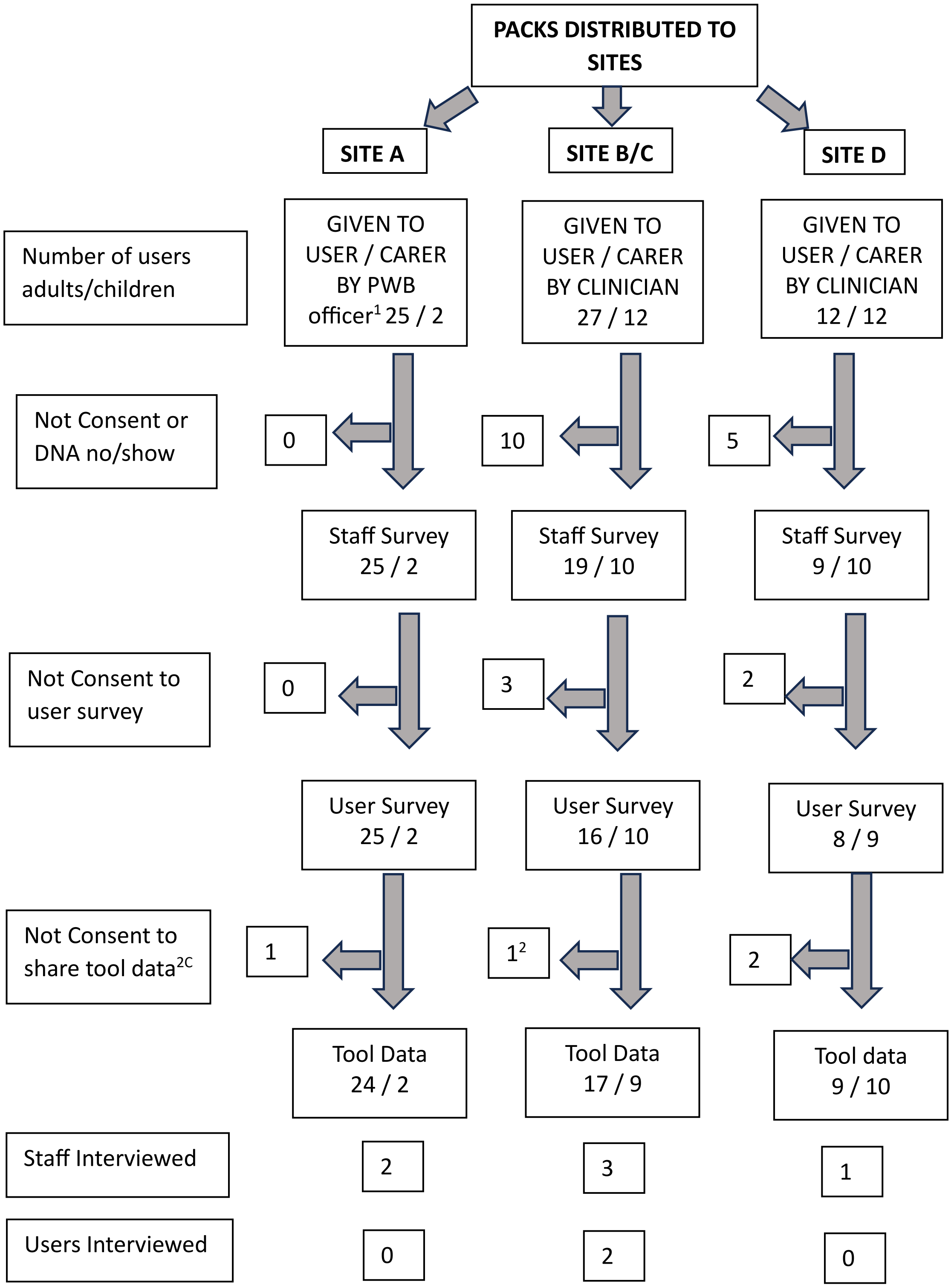

Notes
[1]Personal Wheelchair Budget co-ordinator at this site
[2]One consented to share tool data but not the survey
