# Supplementary material for: Use of patient-centred outcome measures alongside the personal wheelchair budget process in NHS England: A mixed methods approach to exploring the staff and service user experience of using the WATCh and WATCh-Ad
Source: PLoS One. 2025 Jan 10;20(1):e0312967. doi: 10.1371/journal.pone.0312967 (PMC11723643; doi:10.1371/journal.pone.0312967)
Supplement: S5 File — (PDF) [file pone.0312967.s005.pdf]

SLIDE 1: TIME TO COMPLETE WATCH OR WATCH-AD – STAFF VERSUS USER SURVEYS

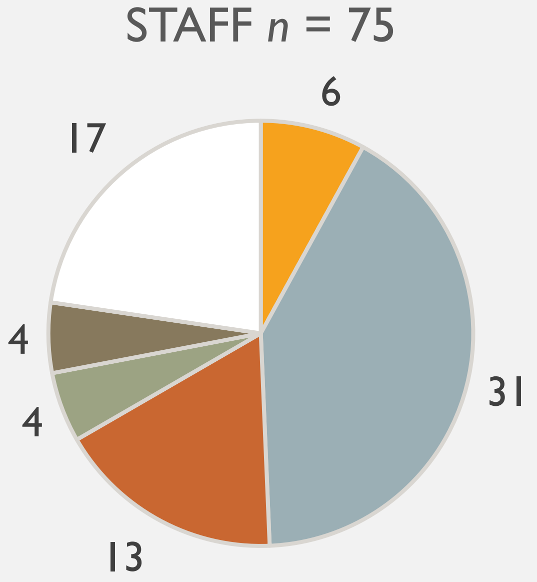

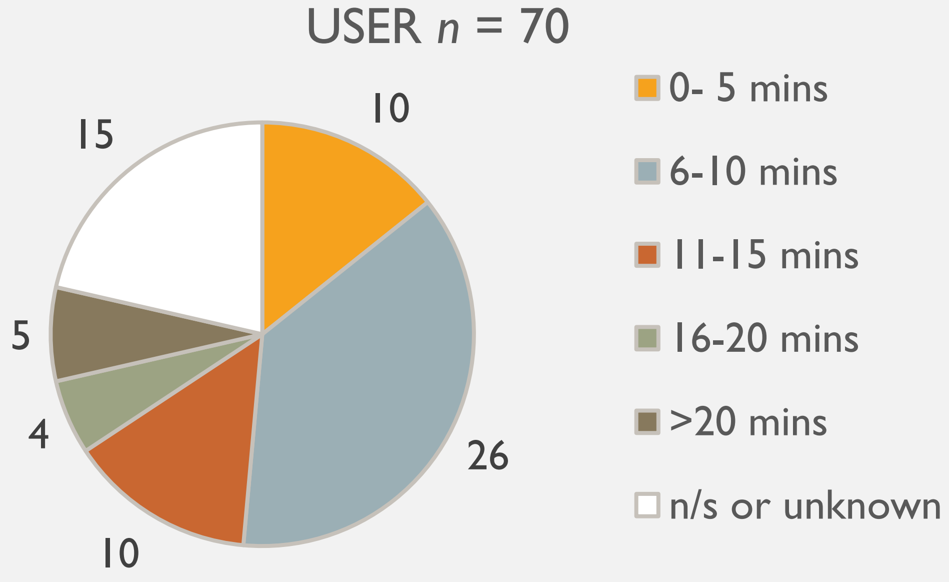

Mean stated = 12.2 minutes (n = 58)

Mean stated = 12.2 minutes (n = 55)

Mean time reported by 11 users where staff had not reported/ did not know = 11.1 mins

SLIDE 2: PERSON COMPLETING THE WATCH OR WATCH-AD

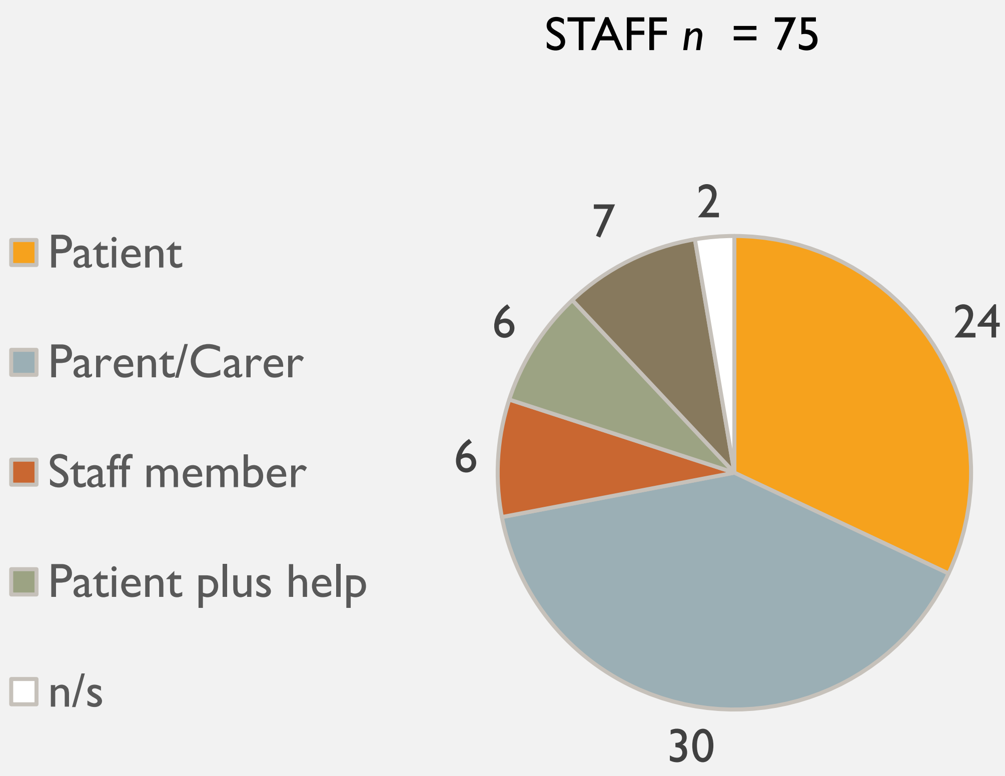

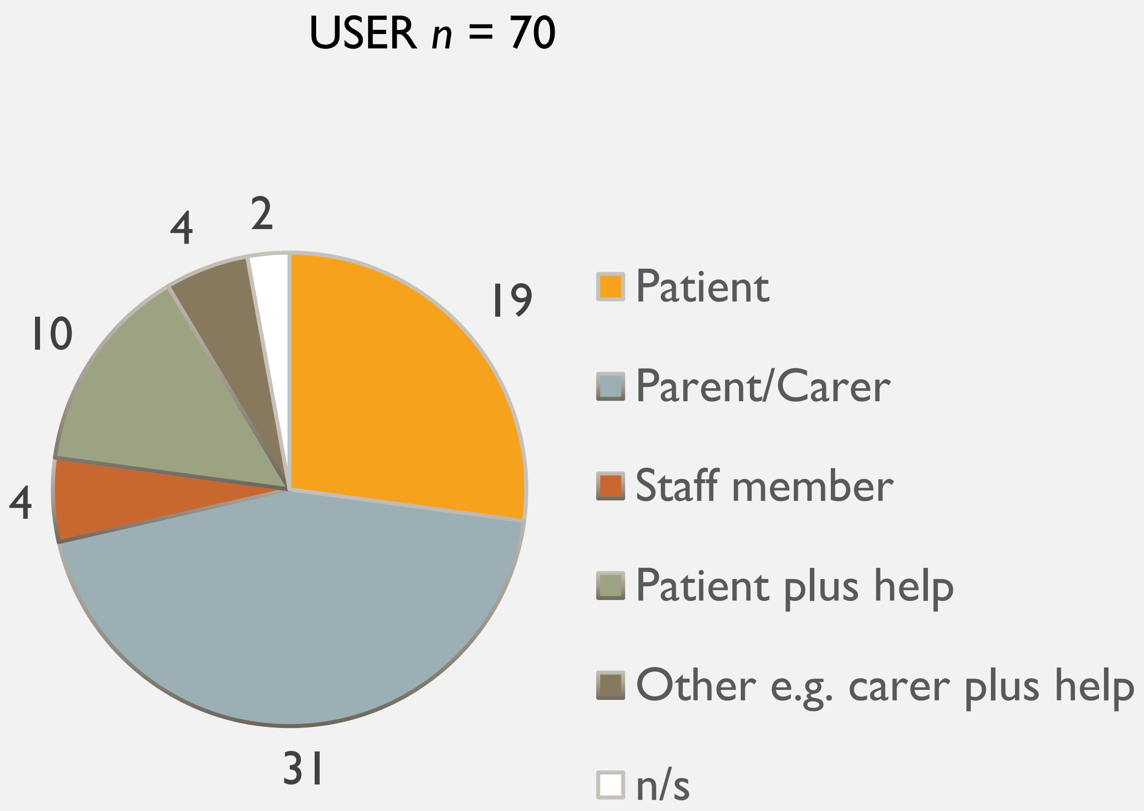

SLIDE 3: STAFF NOTING USER PROBLEMS WITH USING THE TOOLS AND ABILITY TO RESOLVE

'Any problems or questions?' n=75

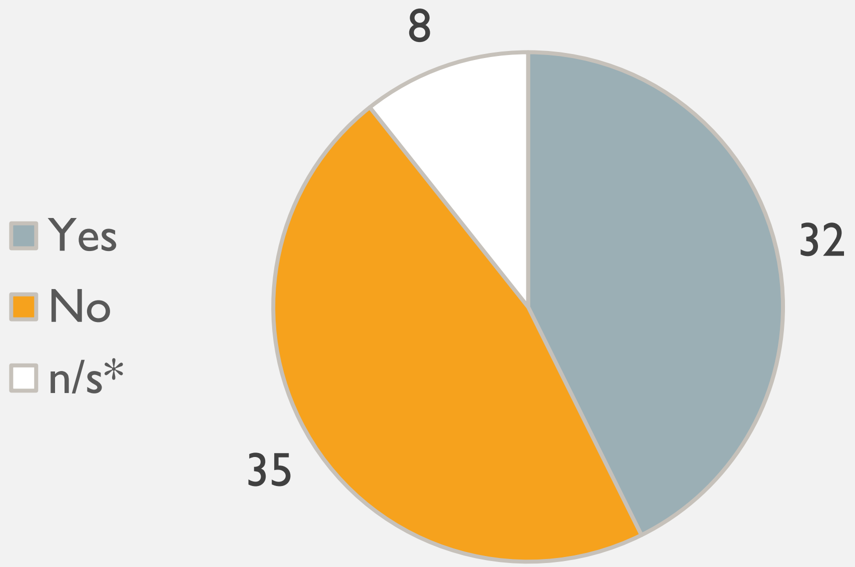

Ability to resolve *n* = 40

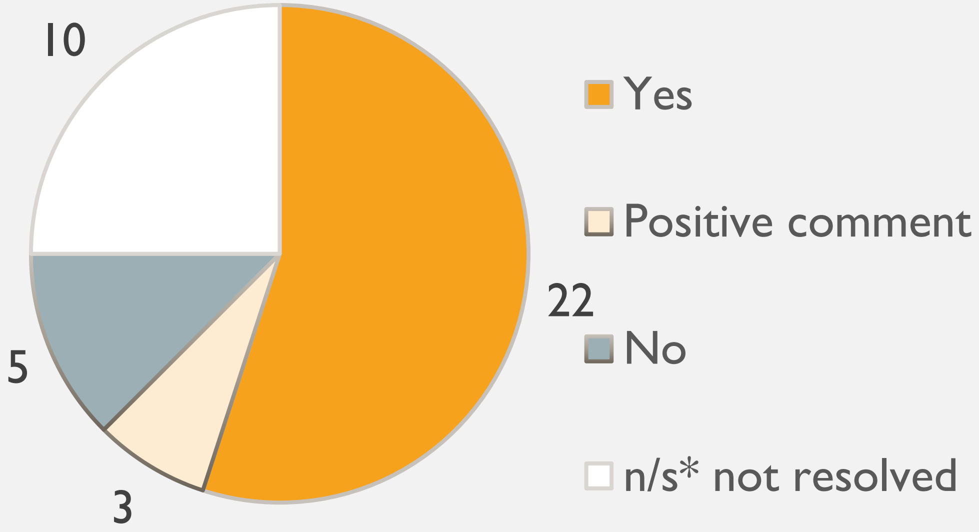

*3 did not go on to an assessment, 5 n/s but comments cited problems

SLIDE 4: USER OR CARER VIEW OF PROBLEMS WITH THE WATCH OR WATCH-AD TOOL

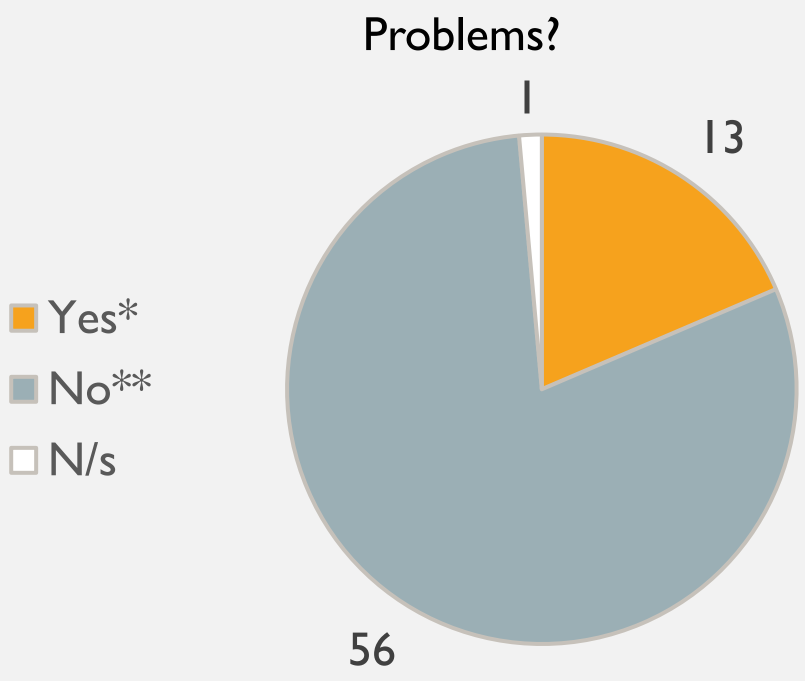

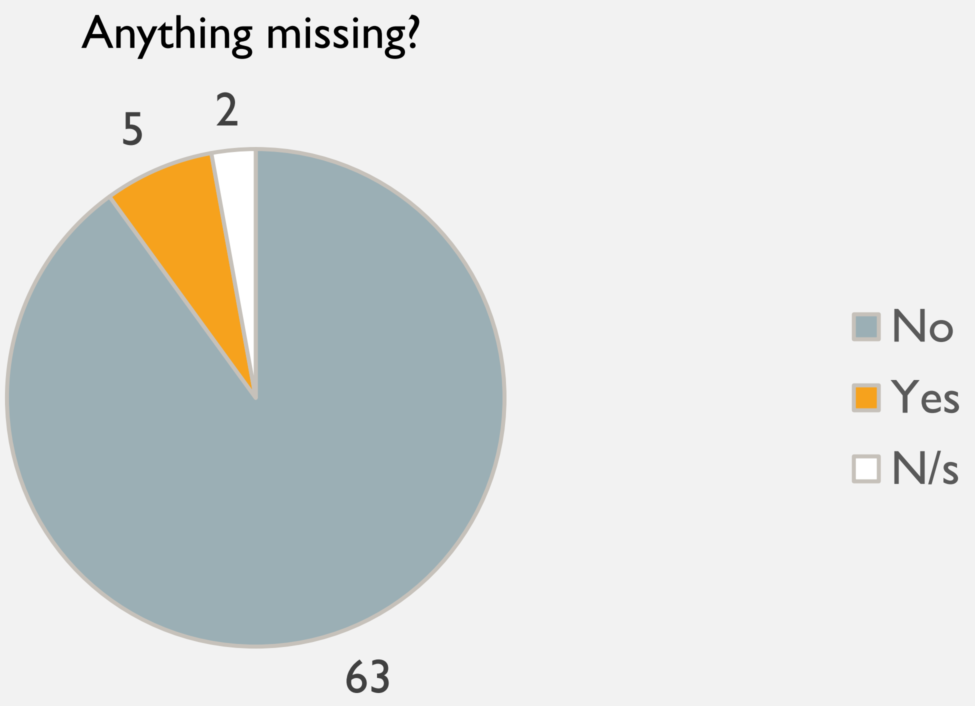

*Only 5 also felt not easy to understand **2 commented not easy to understand

SLIDE 5 COMPARISON OF STAFF AND USER RATING OF USEFULNESS AND HELPFULNESS

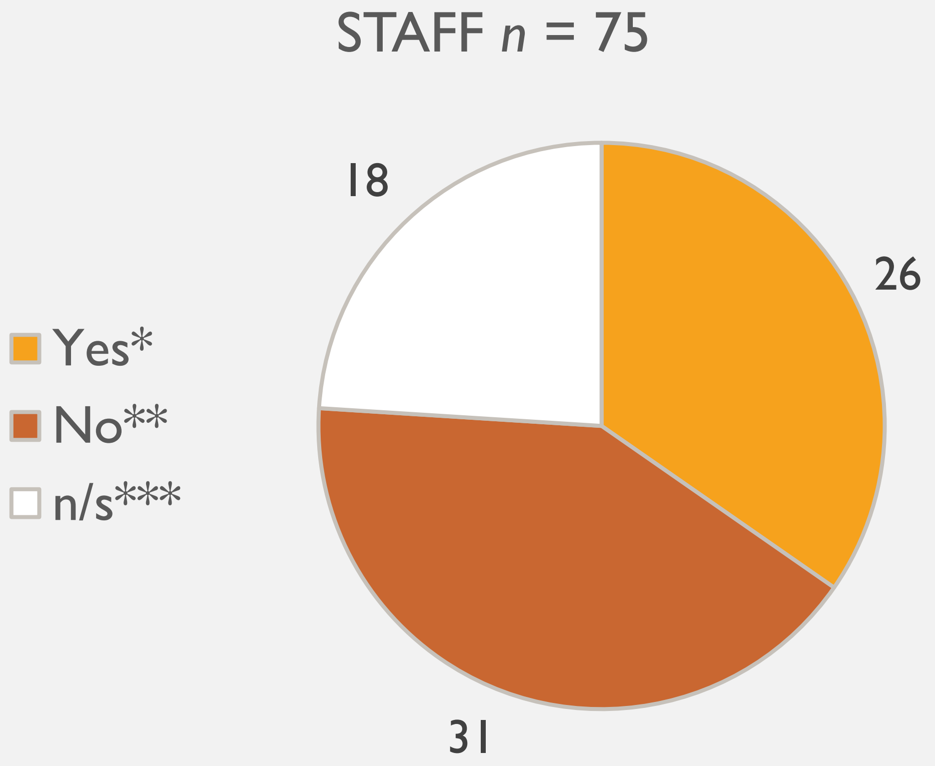

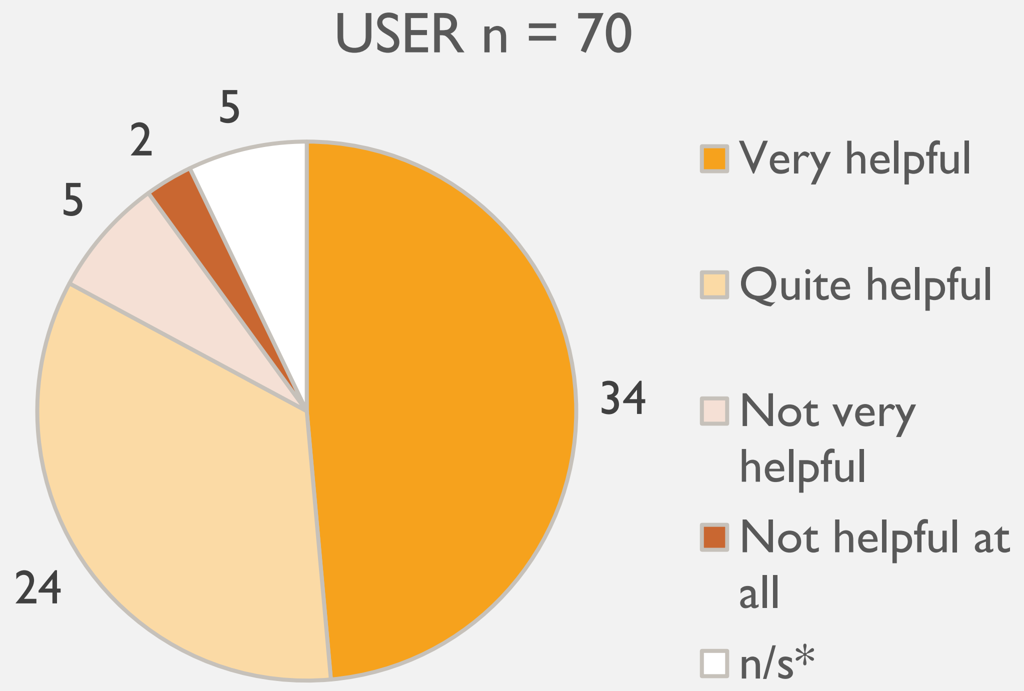

NB: In comparison:
*Only 3 users felt it was not very or not helpful at all
**18 users felt very helpful or quite helpful
***17 users felt very helpful or quite helpful.

*Staff reported all 5 as not useful

SLIDE 6: STAFF PERCEPTIONS OF THE IMPACT OF USING THE TOOLS ON PRESCRIPTION - ADULTS V CHILDREN UNDER 16

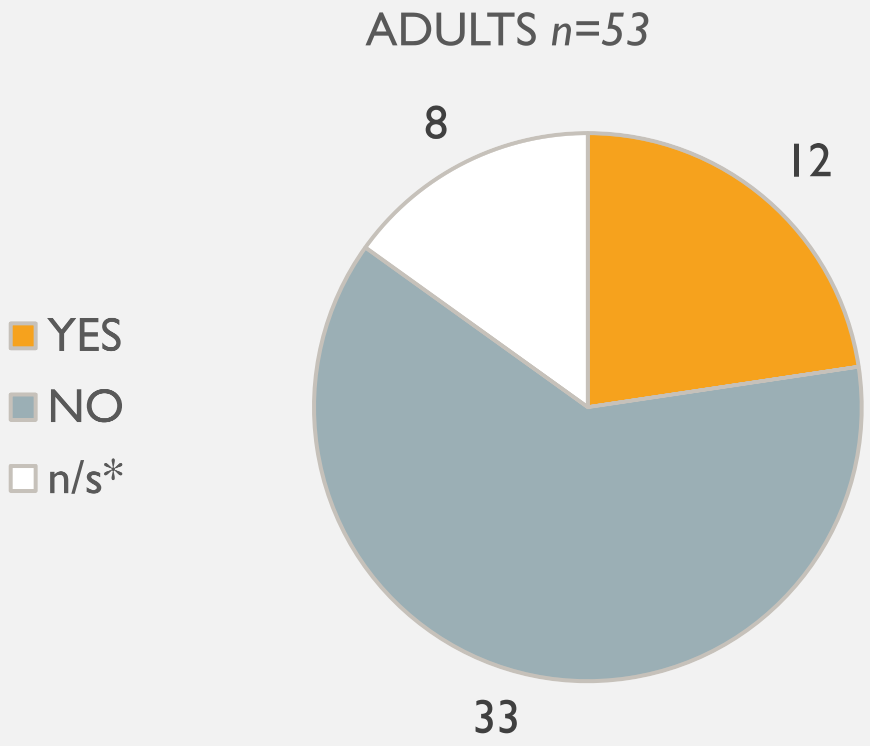

3 n/s in Site A were not assessed

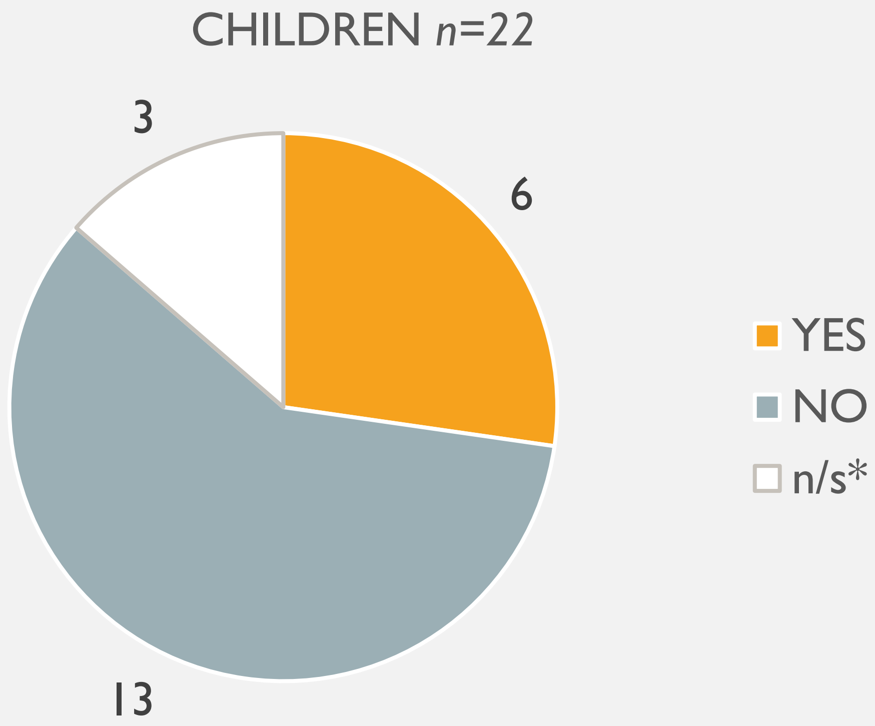

*likely to have been 'No' from other responses

SLIDE 7: ADDITIONAL TIME SPENT BY STAFF USING THE TOOLS IN THE ASSESSMENT – ASSESSOR ONLY AND ANY ADDITIONAL STAFF

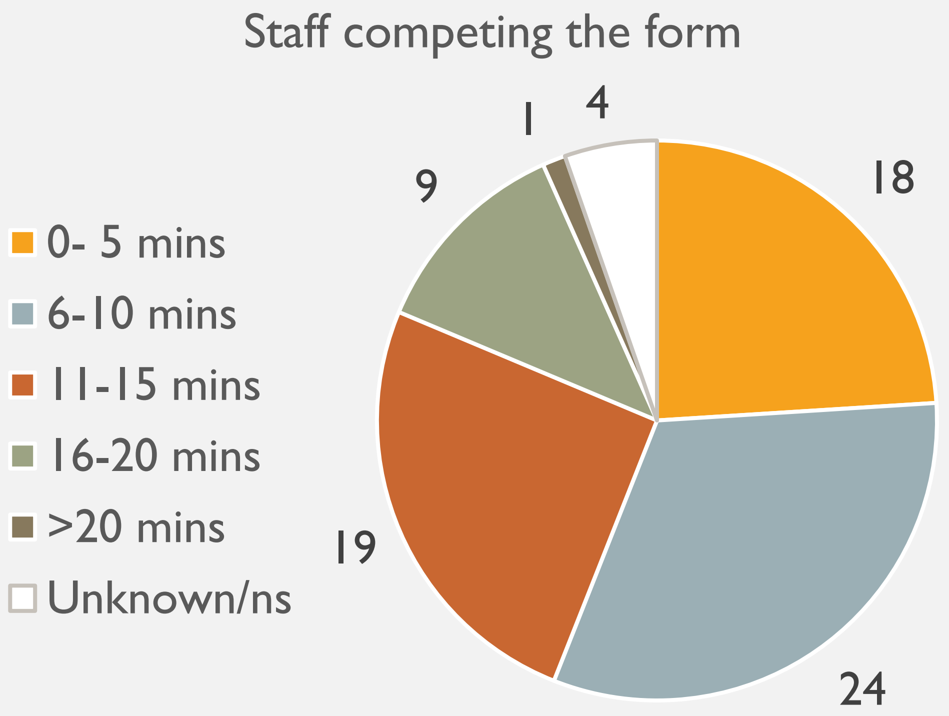

Mean stated = 11.4 minutes (*n* = 71)

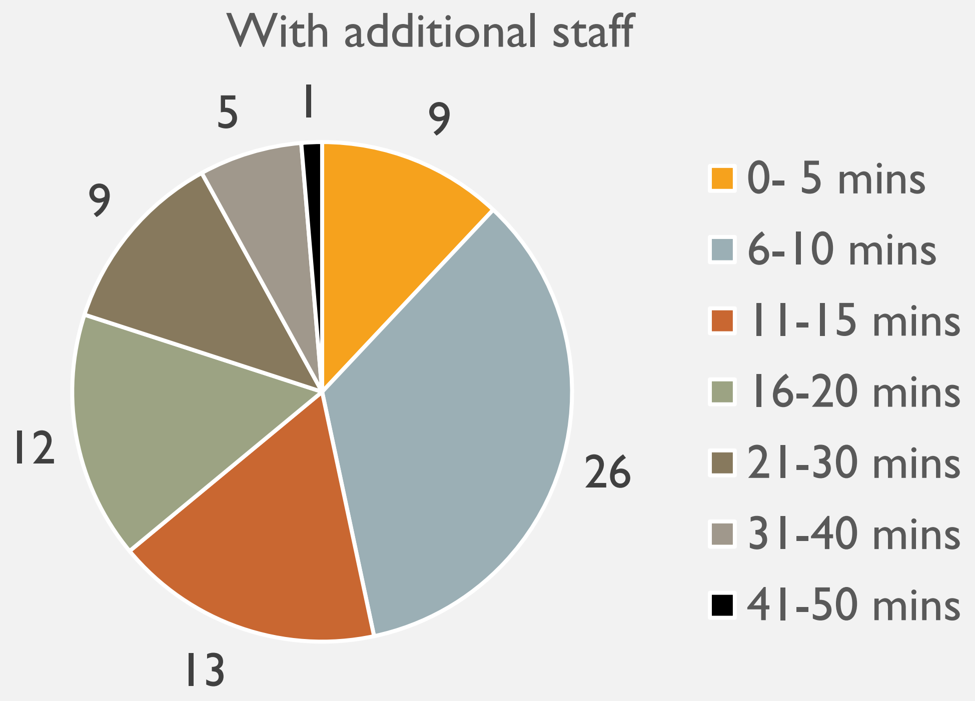

Mean stated = 16.5 minutes (*n* = 71)

SLIDE 8: EQUIPMENT PWB OPTION SELECTED

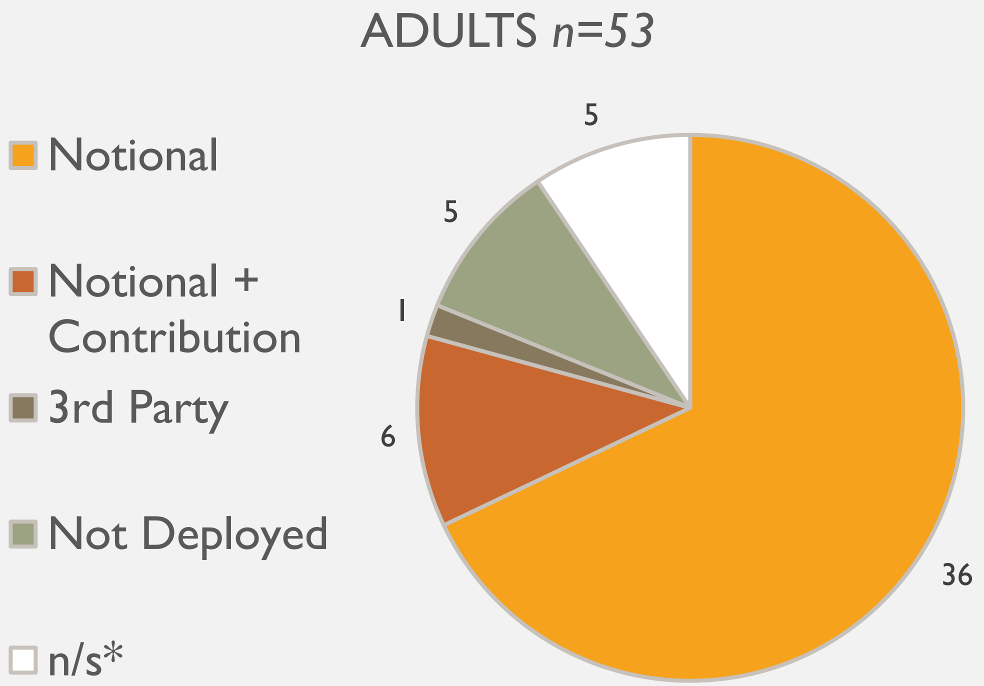

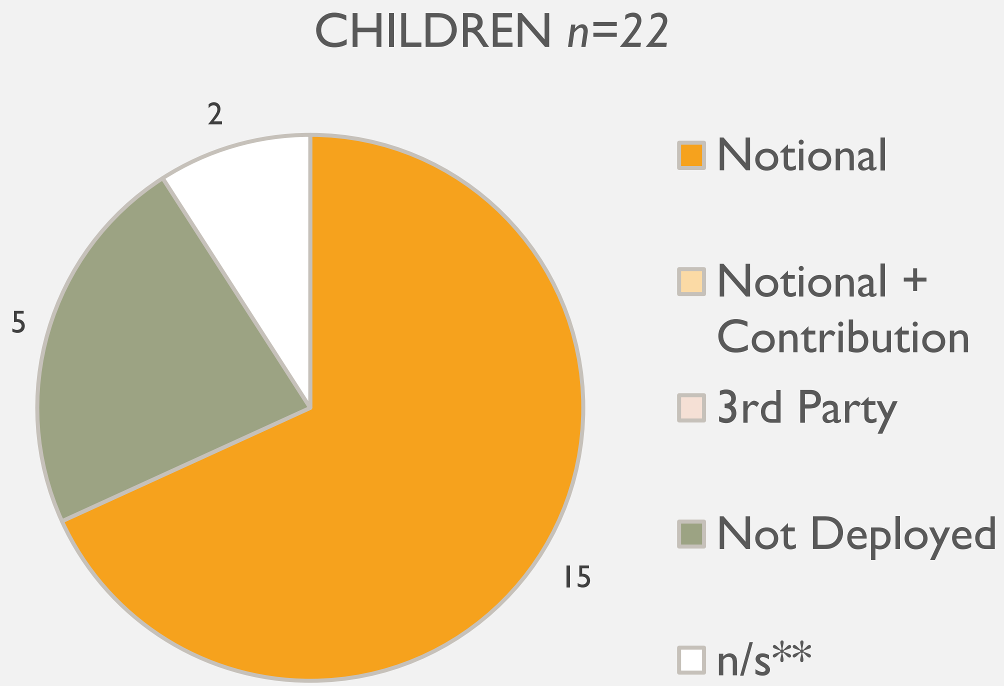

3 n/s in Site A were not assessed

1 n/s in Site D ‘unsure’
