# Supplementary material for: Use of patient-centred outcome measures alongside the personal wheelchair budget process in NHS England: A mixed methods approach to exploring the staff and service user experience of using the WATCh and WATCh-Ad
Source: PLoS One. 2025 Jan 10;20(1):e0312967. doi: 10.1371/journal.pone.0312967 (PMC11723643; doi:10.1371/journal.pone.0312967)
Supplement: Attachment — Submitted filename: comment from reviewer.pdf [file pone.0312967.s008.pdf]

This study aimed to assess the use of the WATCh and the WATCh-Ad assess for outcome achievement in wheelchair user and service user. This paper is the useful study that can improve the assessment and the PWB service. I think this manuscript should be accepted. However, there are little suggestion to improve the rationale and gap of this study. Specific comment for each section is below:

Method

Page 15 Line 158 : Is the author have the evident about the validity and reliability of WATCh and the WATCh-Ad ? Please present the psychometric properties of the questionnaire. and it can be used as the evidence in discussion part.

Page 19 Line 262 : For quantitative analysis, why this study was not use comparative statistics for analysis score between pretest and follow up period?

Results

Page 24 Line 336-338: Please add percentage after number in the problems that were reported

Discussion

Page 36 Line 617 : In the result section, there are report some of the disadvantage and limitation of WATCh and the WATCh-Ad. Please discuss more information to solve this problem and how to apply in service user.
