# Supplementary material for: Use of patient-centred outcome measures alongside the personal wheelchair budget process in NHS England: A mixed methods approach to exploring the staff and service user experience of using the WATCh and WATCh-Ad
Source: PLoS One. 2025 Jan 10;20(1):e0312967. doi: 10.1371/journal.pone.0312967 (PMC11723643; doi:10.1371/journal.pone.0312967)
Supplement: Attachment — Submitted filename: Response to Editor and Reviewers.docx [file pone.0312967.s009.docx]

**Response to Editor’s comments**

| **Item** | Comment (abridged) | Response/ Rebuttal |
| --- | --- | --- |
| **1** | Please ensure that your manuscript meets PLOS ONE's style requirements, including those for file naming | Addressed |
| **2** | Consider depositing your raw data in a repository | Participants could be identifiable from the level of detail required within the information due to small number of sites and subjects |
| **3.1** | ‘Funding Information’ and ‘Financial Disclosure’ sections do not match | Addressed |
| **3.2** | Provide the correct grant numbers for the awards you received for your study in the ‘Funding Information’ section | Not applicable - no grant numbers ot Addressed |
| **4** | Please include your updated Competing Interests statement in your cover letter; we will change the online submission form on your behalf. | Provided in cover letter |
| **5** | Please clarify whether this [conference proceeding or publication] was peer-reviewed and formally published. | Addressed in cover letter |
| **6** | Please also provide non-author contact information (phone/email/hyperlink) for a data access committee, ethics committee, or other institutional body to which data requests may be sent. | As Item 2 |
| **7** | Amend your list of authors on the manuscript to ensure that each author is linked to an affiliation | Addressed |
| **8** | Please include your full ethics statement in the ‘Methods’ section of your manuscript file. In your statement, please include the full name of the IRB or ethics committee who approved or waived your study, as well as whether or not you obtained informed written or verbal consent. | Addressed |
| **9** | Please upload a copy of Supporting Information Figure/Table/etc. S5 Dataset which you refer to in your text on page 35 | Provided as S6 and S7 (New S4 flowchart) |
| **10** | 10. Please review your reference list to ensure that it is complete and correct. If you have cited papers that have been retracted, please include the rationale for doing so in the manuscript text, or remove these references and replace them with relevant current references. | Addressed |

**Response to Reviewer 1’s comments**

| **Item** | **Comment (abridged)** | **Addressed/Rebuttal** |
| --- | --- | --- |
| **1** | Method Page 15 Line 158 : Is the author have the evident about the validity and reliability of WATCh and the WATCh-Ad ? Please present the psychometric properties of the questionnaire. and it can be used as the evidence in discussion part. | The WATCh and WATCh-Ad are intended as tools for use by clinicians in their assessment of an individual wheelchair user's needs, along with the user or their carer, based on aspects of their life that could be impacted by their personal circumstances and need for equipment. They are not intended to be used to make comparisons between groups of users, different services or equipment, for which other measures are available; we have attempted to make this clear in the paper (see lines 604-607). The outcomes areas were determined from previous work (see references #7 and #8). |
| **2** | Method Page 19 Line 262 : For quantitative analysis, why this study was not use comparative statistics for analysis score between pretest and follow up period? | The scoring system aims to indicate any change in the level of satisfaction before and after receipt of the equipment for an individual service user. They are not appropriate for use to make comparisons between groups of users, different services or equipment, for which other measures are available; we have attempted to make this clear in the paper (see lines 604-607). For this reason and because of the small number of users receiving their equipment in the time period of the study it was not felt appropriate to carry out statistical analysis, but to present the scoring data. Further comparative analysis is beyond the scope of this study and would not be in-keeping with the purpose of the tools. |
| **3.1** | Results Page 24 Line 336-338: Please add percentage after number in the problems that were reported | Addressed |
| **3.2** | Discussion Page 36 Line 617 : In the result section, there are report some of the disadvantage and limitation of WATCh and the WATCh-Ad. Please discuss more information to solve this problem and how to apply in service user. | Lines added into discussion (new lines 639 – 646) and conclusion (line 736) |

**Response to Reviewer 2’s comments**

| **Item** | **Comment (abridged)** | **Addressed/Rebuttal** |
| --- | --- | --- |
| 1 | Part introduction : To ensure that your outcomes aligns seamlessly with your aim. Should add any specific details regarding the desired outcomes and how they will be measured. | This study was not aiming to obtain desired outcomes but to gain insight into the usability. Last paragraph of introduction has been revised to reflect this (lines 45 – 46). |
| 2 | Part Method - The incorporation of a flowchart is recommended to facilitate a clearer understanding of the study protocol among readers. | Flowchart added as new Supplementary File 4 |
| 3 | - Is there a potential impact on the study results by eliminating certain groups of samples? It's important to note that samples from each location represent distinct populations, and their exclusion may have implications for the overall findings. | No groups of samples were omitted - the differences in numbers of staff surveys, user surveys and user tool data analysed was where data was not available or participants did not consent to use of particular data. We therefore believe that this comment is beyond the remit of the study. |
| 4 | - Comprehensive information about data analysis, with a focus on addressing rigor and trustworthiness issues, should be included. | As noted in 1) the study aimed to obtain descriptive statistics on acceptability of use of the tool use among users and staff to obtain information on users' key outcomes and score before and after receipt of their equipment. We therefore feel that the request to report comprehensive information about data analysis, focussing on rigor and trustworthiness, is beyond the stated remit of this study |
