# Supplementary material for: Use of patient-centred outcome measures alongside the personal wheelchair budget process in NHS England: A mixed methods approach to exploring the staff and service user experience of using the WATCh and WATCh-Ad
Source: PLoS One. 2025 Jan 10;20(1):e0312967. doi: 10.1371/journal.pone.0312967 (PMC11723643; doi:10.1371/journal.pone.0312967)
Supplement: Attachment — Submitted filename: Response to Editor and Reviewers 20240628.docx [file pone.0312967.s010.docx]

**Response to Editor’s comments**

| **Item** | **Comment (abridged)** | **Response/ Rebuttal** |
| --- | --- | --- |
| **1** | If you have cited papers that have been retracted, please include the rationale for doing so in the manuscript text, or remove these references and replace them with relevant current references. | Assuming this relates to comments from Reviewer 3, the additional work they refer to is already is already cited – see rebuttal #1 |

**Response to Reviewer 3’s comments**

| **Item** | **Comment (abridged)** | **Addressed/Rebuttal** |
| --- | --- | --- |
| **1** | Introduction: The manuscript lists several mobility assessment tools however, there is little comparison on how these tools measure satisfaction related to mobility. For children, the FMA-FC tool was developed in early 2018. The MOBQOL is developed for QOL assessment. Please include these tools in the review and synthesize why the WATCH is piloted in this study than other validated tools. | The MoBQoL and FMA-FC are both already cited as references 8 and 16 respectively, however the authors thank the reviewer for highlighting that the FMA has been adapted for use with children as the FMA-FC, and that further explanation is required to explain why the WATCh tools offer a different approach to all those listed. New statements added at lines 95-96, 115, 138-142. |
| **2** | Psychometric properties of the tool: Please verify if this tool has undergone test-retest reliability testing. If not, please note it as a limitation. This means the responses to the tool outcome items will vary across times for a user and across users with same outcome levels. | One of the aims of the WATCh and WATCh-Ad tools is to obtain a baseline measure of satisfaction with the aspects of life highlighted as of most importance to the wheelchair user. This is then compared with a re-score of satisfaction after use of the equipment provided subsequently. Therefore it would be anticipated that the scoring should change with time, hopefully improved, if the equipment has helped meet the user's needs |
| **3** | Main Aim: As stated on line 134, the main study aim is to compare satisfaction ratings before and after. The question is HOW? Please provide methodology and statistical testing for the same. | The authors thank the reviewer for the comment and have explained that the scoring for the WATCh and WATCh-Ad tools is within-patient only, i.e. the clinician is only looking at any numerical change between assessment and post-equipment scores for that patient. A new statement is added at line 91-93 and summary findings presented at lines 477-480. As the tools are not intended to provide scoring between patients or to be used to compare performance of different equipment or different service providers etc., as each patient's situations and needs are unique. Therefore formal statistical analysis is inappropriate. |
| **4** | Table 1 should be in results. The COVID-19 related content does not seem relevant. | Table 1 is included within the Methods section to show the features of the sites agreeing to participate, which are not results of the study per se. The authors feel that the Covid section is relevant to explain the background to carrying out a study involving visits to clinics. |
| **5** | Line 266: Please describe the method to transform qualitative data into themes. | The manuscript states "Qualitative data obtained through the surveys and the interviews was transcribed and analysed for emerging themes assisted by use of a Microsoft Excel spreadsheet." |
| **6** | The way results are arranged under subheadings, could you arrange the methods and analyses. | The results have been presented in this way for easy comparison of findings between the users and staff. A statement has been added at lines 279-280 under Analysis "Results are presented by the type of information obtained in order to compare between staff and service users." |
| **7** | Line 330: separate the mean times for the two cohorts. | The mean time was the same for staff and users at 12.2 minutes each. The range of responses has been added separately for staff and users at lines 344-345 |
| **8** | Discussion: Please begin this section with the highlights of the study - usability and acceptance of the tool. Was your main aim addressed? The time assessment noted in first parah can follow. | New paragraph inserted. |
| **9** | The validity text from 649-670 is redundant as far as the main aim is concerned. | The authors would like to maintain the inclusion of this to avoid challenges that validity has not been addressed |
