# Supplementary material for: Use of patient-centred outcome measures alongside the personal wheelchair budget process in NHS England: A mixed methods approach to exploring the staff and service user experience of using the WATCh and WATCh-Ad
Source: PLoS One. 2025 Jan 10;20(1):e0312967. doi: 10.1371/journal.pone.0312967 (PMC11723643; doi:10.1371/journal.pone.0312967)
Supplement: Attachment — Submitted filename: Response to Editor and Reviewers 20240902.docx [file pone.0312967.s011.docx]

**Response to Editor’s comments**

| **Item** | **Comment (abridged)** | **Response/ Rebuttal** |
| --- | --- | --- |
| **1** | If you have cited papers that have been retracted, please include the rationale for doing so in the manuscript text, or remove these references and replace them with relevant current references. | N/A |

**Response to Reviewer 3’s comments**

| **Item** | **Comment** | **Addressed/Rebuttal** |
| --- | --- | --- |
| **6** | The second comment is about test-retest reliability which is a limitation of the tool before you perform usability testing in this study. I understand the response but that does not verify test-retest unfortunately. The third comment is about the lack of statistical analysis to test your aim which is not in your abstract methods and is present in the manuscript. See Aims: Our aim was to assess outcome achievement at the level of the patient, referred to here as the service user, by comparison of satisfaction ratings with their key outcome areas before and after provision of equipment, and identify aspects of use of the tools important to them and the service provider in order to maximise wider implementation. So, you are comparing satisfaction. In your Analysis section there is not statistical test for that. In the results line 466 onwards and Fig. 3, you simply say higher or lower scores but you want to test for statistical significance as that is your main aim. | Thank you for clarifying your comments.  Due to the way the tool is implemented with the user through discussion with the service provider, it was considered impractical to carry out a test-retest for the pre- and/or post- provision of equipment phases. Your comments have been noted and we have now added this limitation within the Discussion.  Due to the nature of the study, the protocol planned for descriptive statistics. We do not state within the manuscript that a formal statistical analysis was performed. In response to your comment, we have further clarified in the results section of the abstract that results are simply presented graphically. |
